# Axonal Stimulations With a Higher Frequency Generate More Randomness in Neuronal Firing Rather Than Increase Firing Rates in Rat Hippocampus

**DOI:** 10.3389/fnins.2018.00783

**Published:** 2018-10-24

**Authors:** Zhaoxiang Wang, Zhouyan Feng, Xuefeng Wei

**Affiliations:** ^1^Key Lab of Biomedical Engineering for Education Ministry, College of Biomedical Engineering & Instrument Science, Zhejiang University, Hangzhou, China; ^2^Department of Biomedical Engineering, The College of New Jersey, Ewing, NJ, United States

**Keywords:** high frequency stimulation, unit spikes, firing rate, randomness, desynchronization, electrical energy

## Abstract

Deep brain stimulation (DBS) has been used for treating many brain disorders. Clinical applications of DBS commonly require high-frequency stimulations (HFS, ∼100 Hz) of electrical pulses to obtain therapeutic efficacy. It is not clear whether the electrical energy of HFS functions other than generating firing of action potentials in neuronal elements. To address the question, we investigated the reactions of downstream neurons to pulse sequences with a frequency in the range 50–200 Hz at afferent axon fibers in the hippocampal CA1 region of anesthetized rats. The results show that the mean rates of neuronal firing induced by axonal HFS were similar even for an up to fourfold difference (200:50) in the number and thereby in the energy of electrical pulses delivered. However, HFS with a higher pulse frequency (100 or 200 Hz) generated more randomness in the firing pattern of neurons than a lower pulse frequency (50 Hz), which were quantitatively evaluated by the significant changes of two indexes, namely, the peak coefficients and the duty ratios of excitatory phase of neuronal firing, induced by different frequencies (50–200 Hz). The findings indicate that a large portion of the HFS energy might function to generate a desynchronization effect through a possible mechanism of intermittent depolarization block of neuronal membranes. The present study addresses the demand of high frequency for generating HFS-induced desynchronization in neuronal activity, which may play important roles in DBS therapy.

## Introduction

Deep brain stimulation (DBS) is an established therapy for treating motor disorders such as Parkinson’s disease and essential tremor (Cury et al., 2017). Due to its fast and reversible actions, as well as fewer side effects than pharmacological treatments, DBS therapy has also shown promise for treating other neurological diseases such as epilepsy, depression, and Alzheimer’s disease (Laxton et al., 2010; Kennedy et al., 2011; Vonck et al., 2013). However, the precise mechanisms of DBS have not yet been determined, limiting the development and informed application of DBS treatment to various neurological disorders (Udupa and Chen, 2015; Florence et al., 2016). One of the key questions desired to be addressed is why DBS requires a high-frequency persistent pulse stimulation to obtain therapeutic efficacy in most treatments (Herrington et al., 2016). Because stimulation frequency is an important determinant of the consumption of electrical energy and the battery life-span of implantable pulse generators, answers to the question are important for the advancement of DBS therapy.

Clinical investigations have shown that a pulse frequency higher than 90 Hz is effective, whereas a pulse frequency lower than 60 Hz is ineffective or even worsens the symptoms in treating tremors by DBS (Birdno and Grill, 2008; McConnell et al., 2012). Additionally, high-frequency stimulation (HFS) (around 130 Hz) has been shown to be more effective than low-frequency stimulation in treating epilepsy (Jobst, 2010; Vonck et al., 2013). Thus, regular DBS therapy utilizes HFSs (commonly above 100 Hz) of electrical pulses in treating most brain disorders.

According to biophysical theory of excitable cells, a sequence of stimulation pulses could induce neuronal firing by transferring electrical energy into “neuronal energy” by depolarizing membranes to generate action potentials and to transmit excitation signals. Therefore, it seems reasonable to speculate that more electrical energy delivered by stimulations of a higher frequency would generate more neuronal firing. However, over-delivery of electrical energy by intensive HFS can generate a depolarization block of voltage-gated channels in neuronal membranes thereby preventing continuous firing of action potentials (Benazzouz et al., 1995; Beurrier et al., 2001). Therefore, the stimulated neurons (or neuronal elements) could only generate action potentials with a frequency far lower than the frequency of HFS (Hashimoto et al., 2003; Hamani and Temel, 2012; Florence et al., 2016; Feng et al., 2017). Such a low firing rate could be achieved by a lower stimulation frequency that consumes less electrical energy without inducing depolarization block. Then, what is the role of HFS that consumes more electrical energy?

Hypotheses on the mechanisms of DBS originally focused on whether the targeted nuclei are being excited or suppressed by HFS (Vitek, 2002; Florence et al., 2016), but the focus has shifted from excitability to desynchronization in recent years (Medeiros and Moraes, 2014; Popovych and Tass, 2014). Increase of synchronous bursting and rhythmic oscillation of large neuronal populations are associated with many brain diseases such as motor disorders and epilepsy (Gatev et al., 2006; Rampp and Stefan, 2006; Hammond et al., 2007; Jiruska et al., 2013). Studies have shown that HFS utilized by DBS can reduce pathological oscillations and synchronous activity of target neurons (Wingeier et al., 2006; Deniau et al., 2010; Eusebio et al., 2011; Medeiros and Moraes, 2014). Therapeutic effects of DBS may be attributed to a desynchronization effect rather than a rate change of neuronal firing (Hashimoto et al., 2003; McCairn and Turner, 2009). Therefore, we hypothesize that HFS with a higher frequency could generate more randomness in neuronal firing thereby resulting in a desynchronization effect of DBS.

To test the hypothesis, we investigated the reactions of downstream neurons to pulse stimulation with different frequencies (50–200 Hz) at afferent axon fibers in the hippocampal CA1 region of anaesthetized rats. Axons are more prone to be excited by extracellular pulses of HFS than other structure elements of neurons (Ranck, 1975; Nowak and Bullier, 1998; Johnson and McIntyre, 2008). The outputs of axonal HFS can spread widely through projections of axonal fibers (Nowak and Bullier, 1998), which may play important roles in DBS therapy (Girgis and Miller, 2016; Herrington et al., 2016). Therefore, the present study of axonal stimulation could reveal important mechanisms of DBS and especially provide a new explanation for the demand of high frequency pulses in effective DBS.

We conducted the investigation in hippocampal region because the clear lamellar organizations of neuronal structures in hippocampus facilitate the manipulation of stimulation and recording *in vivo* (Andersen et al., 2000, 2007). In addition, hippocampus *per se* is a potential target for the treatments of brain disorders such as epilepsy and Alzheimer’s disease (Jobst, 2010; Laxton et al., 2010). Therefore, the results of axonal HFS in hippocampal regions could be valuable in advancing the applications of DBS.

## Materials and Methods

### Animal Surgery

**FIGURE 1:**
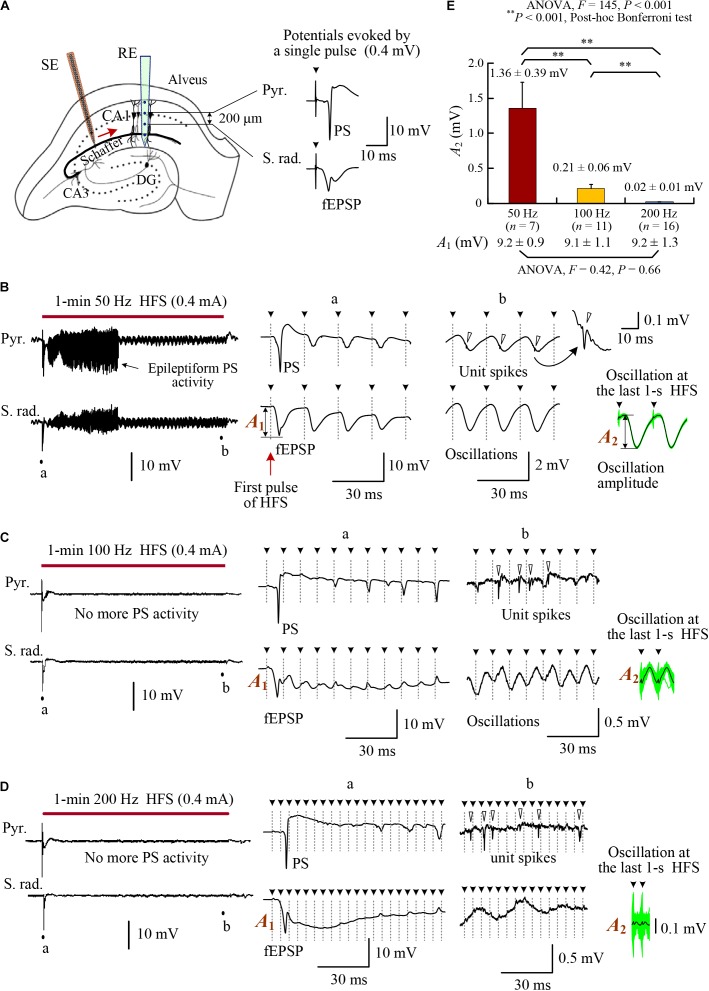
Changes of evoked potentials in the downstream region during HFS of afferent fibers in the rat hippocampal CA1 region. **(A)** Schematic diagram of the locations of recording electrode array (RE) and orthdromic stimulation electrode (SE) in the CA1 region. Two contacts on the recording array separated 0.2 mm were used to collect the potentials in the pyramidal layer (Pyr.) and stratum radiatum (S. rad.), respectively. Typical evoked potentials (PS and fEPSP) by a single pulse are showed on the right. **(B–D)** Neuronal responses to 1-min HFS trains with 50, 100, and 200 Hz pulse frequencies (denoted by the bars). Large PS and fEPSP were evoked by the first stimulation pulse at the onset of HFS. However, in the late HFS period, no more PS potentials appeared in the pyramidal layer and only small oscillations paced pulses in the stratum radiatum. Nevertheless, unit spikes (indicated by hollow arrow heads in the expanded plots) persisted. **(E)** Comparison of the oscillation amplitudes among HFS with different pulse frequencies of 50, 100, and 200 Hz. With similar amplitudes at the onset of HFS (denoted by *A*_1_ and listed on the bottom), the mean oscillation amplitudes at the end of HFS (denoted by *A*_2_) were suppressed more by higher frequencies. The *A*_2_ values were calculated by superposing and averaging the inter-pulse signals in the last 1 s of HFS. See the insets at the lower right of plots **(B–D)**. The green waveforms are superposed signals, and the black curves are average waveforms. Two repeated inter-pulse intervals are drawn for clarity. The solid arrow heads (with dot lines) over the waveforms indicate the removed stimulation artifacts.

All surgical procedures were carried out in accordance with the Guide for the Care and Use of Laboratory Animals (China Ministry of Health). The protocol was approved by the Institutional Animal Care and Use Committee, Zhejiang University, Hangzhou. Twenty adult male Sprague-Dawley rats (321 ± 49 g) were used in this study under anesthesia with urethane. Details of surgery and electrode placements were similar to previous reports (Feng et al., 2013, 2017). Briefly, a 16-channel silicon electrode probe (Model Poly2, NeuroNexus Technologies, United States) was inserted into hippocampal CA1 region to record electrical potentials (Figure 1A). A stimulation electrode, concentric bipolar stainless steel electrode (FHC, Bowdoin, ME, United States) was inserted into Schaffer collaterals of the CA1 region to apply orthodromic stimulation in the upstream of the recording probe. Four neighboring contacts in the recording probe located in pyramidal cell layer were used to collect unit spikes. Another contact located in stratum radiatum, approximate 0.2 mm from the pyramidal layer, was used to collect post-synaptic potentials induced by the stimulation of Schaffer collaterals.

### Recording and Stimulating

Details of recording and stimulating apparatuses have been reported previously (Feng et al., 2017). Briefly, sixteen-channel signals collected by the recording probe were amplified with a frequency band of 0.3–5000 Hz and then sampled at a rate of 20 kHz/channel with 16-bit analog-to-digital conversions. HFS sequences were biphasic current square-pulses with each phase width of 0.1 ms, current intensity of 0.3–0.5 mA, and pulse frequency of 50, 100, or 200 Hz. The duration of pulse sequences was 1 min.

### Data Analysis of Unit Spikes

After removing stimulation artifacts, signals of multiple unit activity (MUA) were obtained by high-pass filtering the raw recording signals with a cut-off frequency of 500 Hz. MUA signals of four neighboring channels located in the pyramidal layer were used to extract single unit activity (SUA) of pyramidal cells and interneurons. See the reference for the processing details of artifact removal, spike detection, and spike sorting (Feng et al., 2017).

To investigate the effects of axonal HFS on the downstream neurons, mean firing rates of MUA and SUA were calculated during the late 30-s period of 1-min HFS and during the baseline (30 s) before HFS as a control. The initial 30-s period of the 1-min HFS was not used to analyze unit spikes, because population spikes (PS) possibly appeared in the period and might contaminate unit spikes, especially during HFS with a lower stimulation frequency of 50 Hz (Feng et al., 2013).

To evaluate the changes of phase-locked relationship between unit spikes and stimulation pulses during HFS with different frequencies, peri-stimulus time histograms (PSTH) were calculated with a bin width of 0.5 ms and a unit of “spike counts per bin.” Two indexes, the peak coefficient and the duty ratio of excitatory phase, were used to quantify the distribution of unit spikes in the inter-pulse intervals of HFS. The definition of the peak coefficient is as follows:

(1) $$\text{Peak coefficient}=\Delta C/C_{\text{ave}}$$

where *ΔC* = (maximum value of PSTH -*C*_ave_), and *C*_ave_ = average value of PSTH over the time span of inter-pulse interval. A large value of peak coefficient indicates a non-uniform PSTH with a sharp peak; otherwise, a peak coefficient ≈0 indicates an even distribution of PSTH.

The definition of duty ratio of excitatory phase is as follows:

(2) $$\text{Duty ratio of excitatory phase}=\frac{\text{number "excitatory" PSTH bins}}{\text{total number of PSTH bins}}\times 100\%$$

where the “excitatory” PSTH bin is defined as a bin with the unit spike counts above 1.2 times of the mean value of baseline recording in the mimic PSTH (see below for the measurement of mimic PSTH). A redundancy of 20% is used in the threshold setting for anti-interference, because the mean coefficient of variation (CV = standard deviation/mean value) of bins in baseline equivalent PSTHs was ∼0.1 and 2-fold CV (∼0.2) would cover ∼95% interferences of a normal distribution. Thus, the duty ratio is the percentage of “excitatory” bins in a PSTH, and describes the concentration of excitatory effects that increase neuronal firing. A small value of duty ratio indicates a narrow phase of excitation or no excitatory phase (duty ratio = 0); otherwise, a duty ratio ≈100% indicates that neuronal firing increases in the entire time span of inter-pulse intervals.

The two indexes together offer a quantification of the phase-lock relationship between neuronal firing and stimulation pulses. A PSTH with a larger peak coefficient and a smaller duty ratio indicates a stronger phase-locked excitatory effect of stimulation on neuronal firing; otherwise, a PSTH with a smaller peak coefficient and a larger duty ratio indicates a more random effect of stimulation on neuronal firing.

For control, mimic PSTHs were calculated from baseline recordings of unit spikes by setting mimic inter-pulse intervals as 20, 10, and 5 ms corresponding to “stimulation” frequencies of 50, 100, and 200 Hz. Average value (spike counts per bin) of baseline mimic-PSTH was used to calculate the duty ratio of excitatory phase in the formula (2).

Additionally, during HFS, coupling ratio between stimulation pulses and single unit spikes was used to evaluate the efficiency of stimulus pulses in inducing action potentials in the downstream neurons:

(3) $$\text{Coupling ratio}=\frac{\text{number of unit spikes}}{\text{number of stimuation pulses}}\;\left(3\right)$$

All statistical data were shown as mean ± standard deviation. Paired *t*-test for two data groups (e.g., HFS group vs. corresponding baseline group) or one-way ANOVA with *post hoc* Bonferroni tests for three data groups were used to determine statistical significances of the differences among data groups.

## Results

### Field Potentials and Unit Activity Evoked by HFS With Different Frequencies

To investigate the effects of afferent stimulation on the downstream CA1 neurons, we examined the evoked potentials both in the pyramidal cell layer (Pyr.) and in the stratum radiatum (S. rad.) during stimulations of Schaffer collaterals (Figure 1A). Similar to previous reports (Feng et al., 2013, 2017), large PS and field excitatory postsynaptic potentials (fEPSP) (i.e., highly synchronous responses of downstream neurons) appeared in the initial periods of HFS sequences with any stimulation frequencies of 50, 100, and 200 Hz (see expanded insets “a” in Figures 1B–D). The duration of epileptiform PS activity was longer for lower stimulation frequency 50 Hz, similar to the previous report (Feng et al., 2013).

Following disappearance of PS activity, in the pyramidal layer, unit spikes persisted without integrating into PS potentials. In the stratum radiatum, small potential oscillations paced with each stimulation pulse, indicating impulses of HFS coming from the afferent fibers (see expanded insets “b” in Figures 1B–D). The amplitudes of oscillations decreased significantly with the increase of HFS frequency (Figure 1E). With similar amplitudes of fEPSP (*A*_1_) at the onset of 1-min HFS sequences, the mean oscillation amplitudes (*A*_2_) of post-synaptic potentials at the last 1 s of HFS were only ∼15, ∼2, and ∼0.2% of *A*_1_ for 50, 100, and 200 Hz stimulations, respectively. The data indicate a frequency-dependent attenuation of the effect of individual pulses on post-synaptic potentials.

**FIGURE 2:**
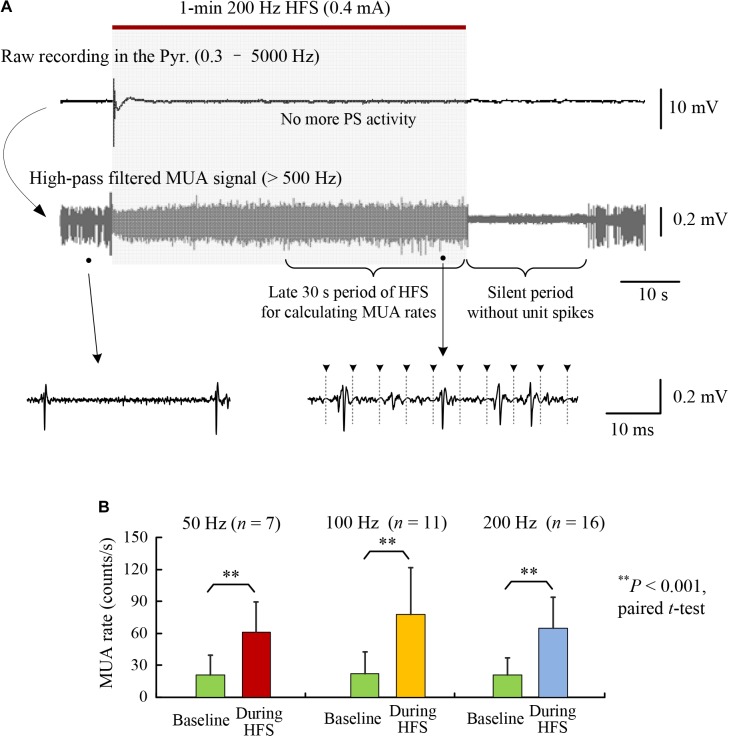
Increase of multiple unit activity (MUA) in the CA1 region during HFS of afferent axons. **(A)** A typical example of neuronal responses to a 1-min train of 200 Hz HFS. The high-pass filtered signal (>500 Hz) shows that the MUA increased during the late period of HFS that was absent of obvious PS activity. **(B)** Comparisons of MUA firing rates between baseline recordings and during late 30-s periods of 1-min HFS with 50, 100, and 200 Hz pulse frequencies. No significant differences existed among the firing rates of the baseline recordings and during HFS, respectively, for the three groups with different frequencies (ANOVA *F* < 1.1, *P* > 0.34).

The stimulations in the late steady-state period of HFS failed to induce large PS events; however, they did increase the firing rates of post-synaptic neurons in the pyramidal layer (Figure 2A). The rates of multiple unit spikes (MUA) during the late 30-s periods of HFS with 50, 100, and 200 Hz were all significantly greater than baseline rates of MUA before HFS. Nevertheless, there was no significant differences among the mean MUA rates during HFS of different frequencies (Figure 2B; ANOVA *F* = 1.1, *P* = 0.34). A silent period (10–30 s) without neuronal firing always appeared immediately after the termination of HFS, indicating that the unit spikes during late HFS must have been induced by the stimulation (Figure 2A).

These results suggest that the difference of pulse frequencies did not cause significant differences in the firing rates of downstream neurons at MUA level, whereas up to a fourfold difference (200:50 frequency) of electrical energy existed among the delivered HFS sequences. The similarity in firing rates posed a difficulty in explaining the frequency-dependent effects of DBS. Nevertheless, the post-synaptic potentials evoked by individual pulses were frequency dependent (Figure 1E), which might change the patterns of neuronal firing. We hypothesize that the extra electrical energy delivered by pulses of a higher frequency would change the firing timing of downstream neurons, rather than their firing rates. Therefore, we next tested the hypothesis by analyzing the distributions of unit spikes in PSTHs during HFS with different frequencies.

### Flattening PSTH Distributions of MUA by Stimulation With a Higher Frequency

**FIGURE 3:**
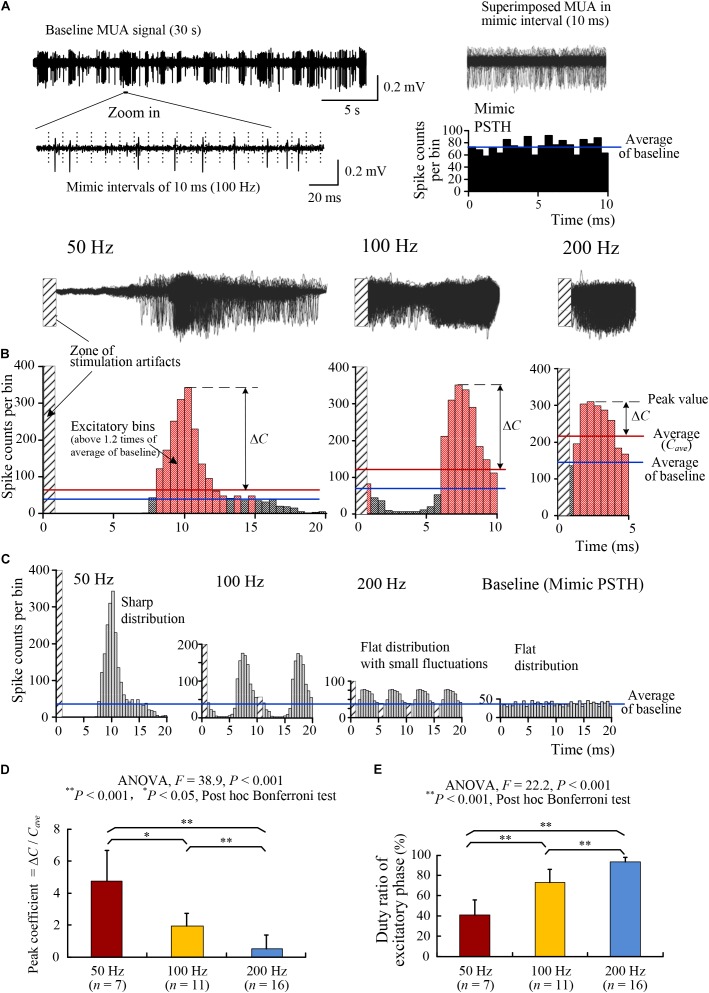
Changes of the PSTH distributions of MUA firing by HFS with various pulse frequencies. **(A)** Making mimic PSTH of MUA firing from 30-s baseline recording for control. *Left*: a typical baseline MUA signal (30 s) was divided by virtual intervals of 10 ms (100 Hz). *Right*: superimposed signals of all 10 ms segments in the 30-s MUA (*up*) and the corresponding mimic PSTH (*down*). The blue line denotes the mean value of PSTH. **(B)** Typical plots of MUA PSTH during late 30-s HFS with 50, 100, and 200 Hz pulse frequencies. *Up*: superimposed signals of all inter-pulse intervals. *Down*: PSTH plots. In the PSTH plots, the red lines and blue lines denote the self-mean values (*C*_ave_) and the baseline mean values, respectively. ΔtextitC is the difference between the peak value and the self-mean. The pink bins of PSTH are with values greater than the baseline mean values (termed as excitatory bins). **(C)** Comparisons of the PSTH distributions under the identical time duration of 20 ms by dividing the PSTH of 100 and 200 Hz into two and four same portions, respectively. **(D,E)** Comparisons of the peak coefficient (Δ*C*/*C*_ave_) and the percentage of excitatory bins (i.e., duty ratio of excitatory phase) among HFS with stimulation frequencies of 50, 100, and 200 Hz.

As a control, mimic PSTH of baseline recording showed that the MUA distribution in mimic inter-pulse intervals was flat, indicating a random distribution of neuronal firing under the situation without stimulation. Figure 3A shows an example of mimic PSTH of 100 Hz HFS for a 30-s segment of baseline recording.

During 50 Hz HFS, the PSTH of MUA was highly non-uniform with a sharp peak at ∼10 ms within the 20 ms inter-pulse intervals. Most unit spikes appeared in the time range of 8–13 ms (Figure 3B, *left*). The non-uniformity of the PSTH decreased with the increase of stimulation frequency (Figure 3B, *middle and right*).

Because the amount of unit spikes are cumulated in different time spans of 20, 10, and 5 ms (4:2:1) for 50-, 100-, and 200-Hz PSTHs, respectively; in order to intuitively compare the PSTH distributions of various frequencies, we divided the PSTHs of 100- and 200-Hz HFS evenly into two and four portions, respectively, and connected the portions together to form a PSTH with 20 ms time span, the same as 50-Hz HFS. Similarly, a mimic PSTH of baseline MUA with 20-ms intervals was also made as a control (Figure 3C). These PSTHs with an identical time span clearly show that the increase of stimulation frequency flattened the distribution of MUA in the inter-pulse intervals, indicating a decrease of phase-locked relationship between the neuronal firing and the HFS pulses.

Statistical data of PSTH suggest that with the increase of stimulation frequency, the mean values of peak coefficient decreased significantly (ANOVA with *post hoc* Bonferroni tests, *P* < 0.001 for comparisons 50 vs. 200 Hz or 100 vs. 200 Hz, *P* < 0.05 for comparison 50 vs. 100 Hz, Figure 3D); meanwhile, the mean values of duty ratio increased significantly (ANOVA with *post hoc* Bonferroni tests, *P* < 0.001 for all three multiple comparisons, Figure 3E).

These data indicate that with similar rates of HFS-evoked firing, stimulations with a lower frequency 50 Hz induced a phase-locked firing, while HFS with higher frequencies 100 and 200 Hz generated a more random pattern of neuronal firing. The extra energy delivered by more pulses of a higher frequency might function to randomize the neuronal firing.

Because MUA signals include unit spikes from a group of neurons, the change of phase-locked relationship revealed from MUA did not necessarily represent the behavior of individual neurons. The dispersion in firing time could be a result of randomized firing time of different neurons, or a result of regular firing time of individual neurons but out of phase from each other. Therefore, we next examined the firing distributions of SUA.

### Flattening PSTH Distributions of SUA by Stimulation With a Higher Frequency

High-frequency stimulation of Schaffer collaterals can excite two types of neurons in the downstream CA1 region: pyramidal cells and interneurons. Analysis of SUA from the two types of neurons showed that, during the late 30-s periods of 1-min HFS with 50, 100, and 200 Hz, the mean firing rates of the neurons all increased significantly comparing to baseline firing (Table 1), although the mean firing rates during HFS of the three different frequencies were not statistically different for both interneurons (ANOVA, *F* = 1.8, *P* = 0.17) and pyramidal cells (ANOVA, *F* = 1.6, *P* = 0.20). Additionally, the coupling ratios between single unit spikes and stimulation pulses decreased significantly with the increase of stimulation frequencies (Table 1). The coupling ratios and firing rates of interneurons were greater than pyramidal cells because of the lower excitation threshold of interneurons (Csicsvari et al., 1998).

**Table 1:** Changes of mean firing rates and coupling ratios of pyramidal cells and interneurons downstream during the late 30-s periods of 1-min 50, 100, and 200 Hz HFS in the Schaffer collaterals of hippocampal CA1 region.

Neuron type	HFS frequency (Hz)	Neuron number	Firing rate in baseline (counts/s)	Firing rate during HFS (counts/s)^a^	Coupling ratio (%)^b^
Interneurons	50	12	5.6 ± 5.0	24.1 ± 15.5	48.3 ± 30.9
	100	17	5.5 ± 5.7	32.5 ± 28.1	32.5 ± 28.5
	200	31	5.9 ± 6.2	20.2 ± 17.8	10.1 ± 8.9
Pyramidal cells	50	26	1.3 ± 1.5	5.1 ± 3.0	10.3 ± 5.9
	100	45	1.2 ± 1.1	6.6 ± 4.3	6.6 ± 4.3
	200	79	1.4 ± 1.3	5.2 ± 4.8	2.6 ± 2.4

*^a^Mean firing rates during HFS of 50, 100, and 200 Hz were all significantly greater than their corresponding firing rates in baseline recordings before HFS (paired t-test, P < 0.001), but were not statistically different from each other for both interneurons (ANOVA, F = 1.8, P = 0.17) and pyramidal cells (ANOVA, F = 1.6, P = 0.20).*

*^b^Mean coupling ratios of the three HFS frequencies were significantly different from each other for both interneurons and pyramidal cells (one-way ANOVA, F > 20, P < 0.001; post hoc Bonferroni tests P < 0.001 for all multiple comparisons: 100 vs. 50 Hz, 200 vs. 50 Hz, and 100 vs. 200 Hz).*

**FIGURE 4:**
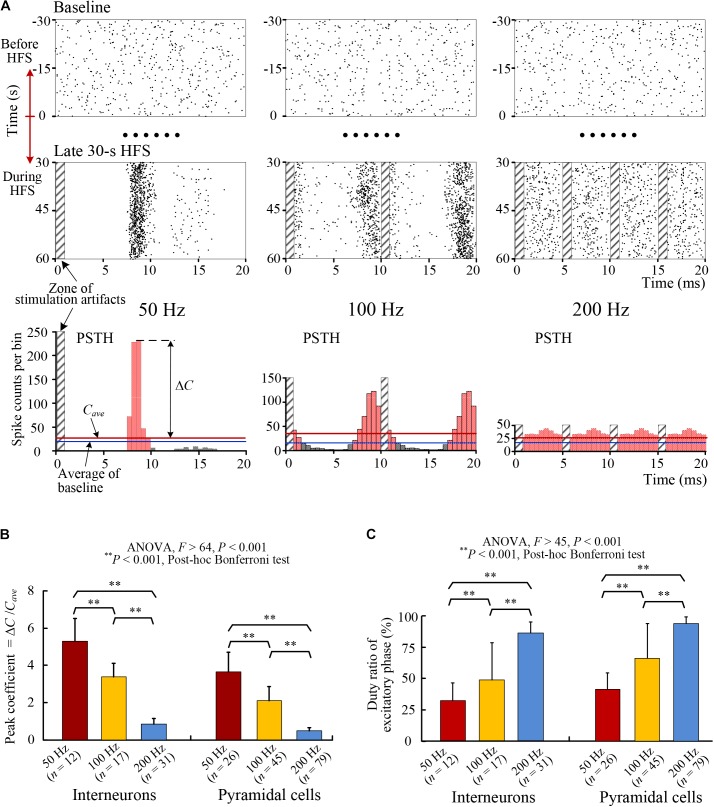
Changes of the PSTH distributions of SUA firing. **(A)** Examples of raster plots and PSTH plots of an interneuron’s firing with HFS of 50, 100, and 200 Hz frequencies. *Top*: raster plots of spikes in baseline before HFS (–30 to 0 s) in mimic interval 20 ms. *Middle*: raster plots of spikes during the late 30-s period of HFS. *Bottom*: PSTH plots during the late period of HFS. The time spans of the three groups of plots were unified to 20 ms to facilitate the comparison of firing rates directly. The PSTH plots of 100 and 200 Hz are duplications of two and four same portions, respectively. **(B,C)** Comparisons of the peak coefficients (Δ*C*/*C*_ave_) and the duty ratio of excitatory phase of PSTH for individual interneurons and pyramidal cells during HFS with different stimulation frequency of 50, 100, and 200 Hz.

Scatter plots of SUA timing in sequential inter-pulse intervals illustrated the distribution of neuronal firing (Figure 4A). For control, during baseline recording before HFS, the unit spikes distributed randomly in mimic intervals of 20 ms (Figure 4A, *Top row*). However, during 50 Hz HFS, the unit spikes distributed centrally in a narrow band except a minor portion of postponed unit spikes (Figure 4A, *Middle row*). Because the coupling ratios were far smaller than 100% (Table 1), even at the stimulation frequency of 50 Hz, the neurons already failed to respond to every stimulation pulse. Even if an action potential was induced, its latency could be lengthened thereby resulting some of the unit firing out of the phase-locked firing timing.

With the increase of stimulation frequencies to 100 and 200 Hz, the coupling ratios decreased further and the distributions of spikes became more and more randomized to finally lose an obvious phase-locked firing timing (Figure 4A, *Middle row*). The PSTHs of SUA with a same interval of 20 ms showed a decrease of peak and an increase of flattening with the increase of stimulation frequency (Figure 4A, *Bottom row*).

Statistical data suggest that for both pyramidal cells and interneurons, the peak coefficients of PSTHs decreased significantly (ANOVA, *P* < 0.001 for all multiple comparisons, Figure 4B) and the duty ratios of PSTHs increased significantly (ANOVA, *P* < 0.001 for all multiple comparisons, Figure 4C) with the increase of HFS frequency.

The obvious peak at around 8–9 ms in the PSTH plots of 50 and 100 Hz indicated the latency of neuronal responses to stimulation pulses. If the interval between stimulation pulses coincided with the peak time of PSTH, the distribution pattern of PSTH would be similar to the PSTH of 100 Hz (corresponding to an inter-pulse interval of 10 ms), except that the peak would overlap more exactly with the zone of stimulation artifacts. However, the firing was not induced by the overlapped stimulation pulse but by the preceding pulse. Additionally, according to the small coupling ratios (Table 1), many of the stimulation pulses failed to induce an action potential.

These results indicate that the changes of PSTH distributions of SUA were similar to those of MUA. The flattening trend of MUA distributions by HFS with higher frequencies should be attributed to a more random firing of individual neurons, but not a collective effect of a population of neurons firing at regular intervals yet mutually out of phase. Moreover, the results suggest that with an adequately high frequency, the axonal HFS was able to generate a temporally random excitatory effect on the downstream neurons instead of a sharp effect phase-locked with stimulation pulses, thereby resulting in a desynchronization effect of DBS.

## Discussion

The major findings in this study include the following: (1) the neuronal firing rates induced by axonal HFS with different frequencies (50, 100, and 200 Hz) were similar despite an up to fourfold difference in the number of stimulation pulses and in the electrical energy delivered by stimulations. (2) Stimulation with a higher pulse frequency generated more randomness in neuronal firing timing instead of increasing firing amount. Possible mechanisms underlying the findings and their clinic implications are discussed below.

### HFS-Induced Axonal Block Might Limit the Increase of Neuronal Firing Rate With Increasing Stimulation Frequency

Previous studies have shown that HFS with a frequency over 50 Hz can partially block the activation of axons in hippocampus and subthalamus *in vitro* and *in vivo* (Jensen and Durand, 2009; Zheng et al., 2011; Feng et al., 2013; Rosenbaum et al., 2014). Simulation studies suggest that the outflow of potassium ions induced by intense excitation of axons may accumulate in the small peri-axonal space, thereby generating a depolarization block on axonal membranes because of inactivation of sodium channels (Bellinger et al., 2008; Liu et al., 2009). Under the situation of partial block of axons, HFS pulses can still generate intermittent impulses to the projecting neurons (Garcia et al., 2003; Jensen and Durand, 2009; Feng et al., 2014, 2017). Based on those previous results, here we might as well focus on the mechanism of HFS-induced axonal failures to explain our first finding: a saturation in the increase of neuronal firing rates with the increase of stimulation frequency, although involvements of other mechanisms, such as failures in synaptic transmissions, cannot be excluded currently.

Axonal block induced by HFS is frequency dependent (Jensen and Durand, 2009; Feng et al., 2013, 2014). Each pulse of HFS with a higher frequency could only generate action potentials in a smaller amount of axons thereby generating smaller field post-synaptic potentials (Figure 1). Nevertheless, stimulations with a higher frequency had more pulses and generated more synaptic inputs to the post-synaptic neurons. The summed excitation of smaller but more inputs could counter balance the excitation from larger but less inputs generating by stimulations with a lower frequency (Feng et al., 2014). Therefore, the mean firing rates of both MUA and SUA did not change significantly within 50–200 Hz frequency range of stimulations (Figure 2 and Table 1). The extra electrical energy delivered by stimulations with a higher frequency could have other functions (e.g., randomizing firing) than increasing firing rates of neurons.

Additionally, despite of no statistical significances, the mean rates of neuronal firing seemed reaching a peak at the middle frequency 100 Hz for both types of neurons. That is, the mean firing rates during 200 Hz stimulation were even smaller than the values during 100 Hz stimulation (Table 1). The decline of firing rates by 200 Hz stimulation is reasonable given that a further higher frequency up to kilohertz can completely block axonal firing in peripheral nerves (McGee et al., 2015).

### HFS With Higher Frequency Generates More Randomness in Neuronal Firing

The second interesting finding of our study is that increasing stimulation frequency from 50 to 200 Hz can weaken the phase-locked relationship between unit spikes and stimulation pulses. The smaller coupling ratio induced by axonal HFS with a higher frequency could cause randomness in neuronal firing. For example, during 200 Hz HFS, the mean coupling ratio was ∼10% for interneurons, indicating an interneuron might fire once following one of every 10 pulses, that is, about 10 firing opportunities could be “randomly” chosen in every 50 ms. By contrast, during 50 Hz HFS, only about two opportunities could be chosen because of the mean coupling ratio was ∼50%, meaning more concentrated (i.e., less random) in firing timing. In addition, the loss of phase-locked firing time in the inter-pulse intervals of a higher frequency stimulation generated additional randomness (Figures 3, 4). Both the decreased coupling ratio and the loss of phase-locked relationship could be caused by the mechanism of HFS-induced axonal block and nonlinear dynamics in the recovery course of the block. Presumably, with a higher frequency (e.g., 200 Hz), stimulated axons could constantly be on the way to repolarize, or to recover from a state of prolonged depolarization caused by continuous inputs of stimuli. More randomness in the timing of neuronal firing could result because of the nonlinear dynamics of membrane repolarization (Hodgkin and Huxley, 1952; Grill, 2015). Therefore, the “extra electric energy” delivered by a higher frequency might bring randomness to the firing timing of neurons through elevating the membrane potentials of stimulated axons.

Additionally, although the random neuronal firing during stimulations with a higher frequency seemed close to baseline firing, it could not be a return to baseline firing because of the “silent period” of tens of seconds without unit spikes immediately following the withdrawal of HFS (Figure 2). The silent period clearly showed that the neuronal firing during HFS was driven by the stimuli, not “spontaneous” baseline firing. Otherwise, the baseline firing should have continued following the withdrawal of HFS.

In summary, axonal HFS of a higher frequency generated more randomness in the firing timing of downstream neurons. With similar total amount of firing, the increase of firing randomness suggests not only a decrease of synchronized firing among different neurons but also a decrease of rhythmic firing of individual neurons. The present study provides a novel viewpoint for revealing the mechanism of frequency-dependent efficacy of DBS.

### Implication to the New Findings of HFS

Synchronized and rhythmic firing events are related to pathological reactions of many brain disorders. For example, increase of synchronous bursts and low frequency oscillations in neurons of the basal ganglia and thalamus accompany motor symptoms of Parkinson’s disease (Birdno and Grill, 2008; Gale et al., 2008). Populations of neurons fire in an excessive and synchronized manner in epileptic seizures (Le Van et al., 2003; Lopes et al., 2003).

Recent studies have suggested that desynchronization of neuronal firing is an important mechanism to the therapeutic effects of DBS (Wilson et al., 2011; McConnell et al., 2012; Medeiros and Moraes, 2014). Effective DBS for treating movement disorders overrides pathological oscillations and synchronous activity by replacing them with HFS-induced patterns of activity (Llinas et al., 1999; Birdno and Grill, 2008). Electrical stimulation therapy for epilepsy control has also utilized a strategy to de-synchronize epileptogenic neural networks (Cota et al., 2009; Medeiros and Moraes, 2014). However, the desynchronization effect of DBS requires a stimulation with a high enough frequency (Brown et al., 2004).

The present study addresses the necessarity of a higher frequency for generating HFS-induced desynchronization. With a pulse frequency over 100 Hz, a large portion of the HFS energy utilized by regular DBS might not aim to generate transmissible signals – action potentials, but to add more randomness in the sequences of action potentials thereby causing desynchronization of firing among neurons. As suggested above, a possible mechanism underlying this function of HFS might be intermittent depolarization block of neuronal membranes. The generation of depolarization block may consume a substantial portion of electrical energy.

## Conclusion

The present study shows that neuronal firing rates induced by HFS at afferent axons are similar for stimulation frequencies of 50–200 Hz with an up to fourfold difference in electrical energy. The extra energy delivered by a higher frequency may function to randomize the neuronal firing to avoid phase-locked firing. Possible mechanism of the findings might be the intermittent block of axonal excitation induced by HFS. The findings provide a novel explanation for the demand of high frequency pulses in effective DBS through the mechanisms of desynchronization and dysrhythmia of neuronal firing.

## Author Contributions

ZF, ZW, and XW designed the experiments and/or interpreted the data. ZW and ZF performed the experiments and analyzed the data. ZW and ZF drafted the manuscript. XW revised the manuscript critically for important intellectual content. All authors approved the final version of the manuscript to be published and agreed to be accountable for all aspects of the manuscript.

## Conflict of Interest Statement

The authors declare that the research was conducted in the absence of any commercial or financial relationships that could be construed as a potential conflict of interest.
